# TgTKL4 Is a Novel Kinase That Plays an Important Role in *Toxoplasma* Morphology and Fitness

**DOI:** 10.1128/msphere.00649-22

**Published:** 2023-02-14

**Authors:** Hilary Montano, Ramu Anandkrishnan, Vern B. Carruthers, Rajshekhar Y. Gaji

**Affiliations:** a Department of Biomedical Sciences and Pathobiology, Virginia-Maryland College of Veterinary Medicine, Blacksburg, Virginia, USA; b Department of Biomedical Sciences, Edward Via College of Osteopathic Medicine, Blacksburg, Virginia, USA; c Department of Microbiology and Immunology, University of Michigan, Ann Arbor, Michigan, USA; University at Buffalo

**Keywords:** *Toxoplasma gondii*, apicomplexan parasites, kinases

## Abstract

Protein kinases of the protozoan parasite Toxoplasma gondii have been shown to play key roles in regulating parasite motility, invasion, replication, egress and survival within the host. The tyrosine kinase-like (TKL) kinase family of proteins are a set of poorly studied kinases that our recent studies have indicated play a critical role in *Toxoplasma* biology. In this study, we focused on TgTKL4, another member of the TKL family that is predicted to confer parasite fitness. Endogenous tagging of TgTKL4 identified it as a temporally oscillating kinase with dynamic localization in the parasite. Gene disruption experiments suggested that TgTKL4 is important for *Toxoplasma* propagation *in vitro*, and loss of this kinase resulted in replication and invasion defects. During parasite division, TgTKL4 expression was limited to the synthesis and mitosis-cytokinesis phases and, interestingly, loss of TgTKL4 led to defects in *Toxoplasma* morphology. Further analysis of the parasite cytoskeleton indicated that the subpellicular microtubules are shorter and more widely spaced in parasites lacking TgTKL4. Although loss of TgTKL4 caused only moderate changes in the gene expression profile, TgTKL4 null mutants exhibited significant changes in their global phospho-proteome, including in proteins that constitute the parasite cytoskeleton. Additionally, mice inoculated intraperitoneally with TgTKL4 knockout parasites showed increased survival rates, suggesting that TgTKL4 plays an important role in acute toxoplasmosis. Together, these findings suggest that TgTKL4 mediates a signaling pathway that regulates parasite morphology and is an important factor required for parasite fitness *in vitro* and *in vivo*.

**IMPORTANCE** Toxoplasma gondii is a protozoan parasite that can cause life-threatening disease in mammals; hence, identifying key factors required for parasite growth and pathogenesis is important to develop novel therapeutics. In this study, we identified and characterized another member of the newly described TKL family, TgTKL4, a cell cycle-regulated kinase. By disrupting TgTKL4, we determined that this kinase is required for normal parasite growth *in vitro* and that loss of this kinase results in parasites with reduced competence in replication and invasion processes. Specifically, *Toxoplasma* parasites lacking TgTKL4 had defects in cytoskeletal arrangement, resulting in parasites with abnormal morphology. Phospho-proteome studies provided further clues that decreased phosphorylation of proteins that constitute the *Toxoplasma* cytoskeleton could be responsible for altered morphology in TgTKL4-deficient parasites. Additionally, loss of TgTKL4 resulted in attenuation of virulence in the animal model, suggesting that TgTKL4 is an important virulence factor. Hence, this study provides a novel insight into the importance of a TgTKL4 as a fitness-determining factor for *Toxoplasma* propagation *in vitro* and pathogenesis *in vivo*.

## INTRODUCTION

Toxoplasma gondii is a protozoan parasite classified within the phylum *Apicomplexa*, which includes important human pathogens, such as *Plasmodium*, which causes malaria, and *Cryptosporidium*, which causes diarrhea in neonates. Humans can acquire *Toxoplasma* infection mainly in three ways: ingestion of food or water contaminated with *Toxoplasma* oocysts, ingestion of contaminated meat that contains parasite cysts, and transplacental transmission during pregnancy (1). Following infection with *Toxoplasma*, the acute form of the parasite (tachyzoite) disseminates quickly into various organs in the host. In response to the subsequent host immune response, the parasite evades elimination by converting into an encysted form (bradyzoite) and establishing a chronic infection that persists through the life of the host (2). In immunocompromised individuals, such as those infected with HIV, blood cancer patients, and those undergoing immunosuppressive therapy during organ transplantation, reactivation of the chronic infection or the establishment of new acute infections can lead to fatal toxoplasmosis (3, 4). Additionally, transmission from the mother to the fetus during pregnancy results in congenital toxoplasmosis, causing severe disease in the offspring, including neurological deficiencies and blindness, sometimes resulting in neonatal death (5). While the available current therapy is effective against the acute stage of infection, it can have toxic side effects and, importantly, it does not treat the chronic form of the disease (2). As a result, there is a strong need for the identification and development of novel therapeutic options to treat *Toxoplasma* infections.

The intracellular lifestyle of *Toxoplasma* begins with the parasite gaining entry into the host cell through an active invasion mechanism (6, 7). Once in the host cell, the parasite is surrounded by a parasitophorous vacuolar membrane within which the parasite replicates through a process known as endodyogeny (8–11). After undergoing multiple rounds of division, the parasites egress from a host cell, which is destroyed in the process (12, 13). Importantly, much of the pathology associated with *Toxoplasma* infection is due to the tissue destruction caused by repeated cycles of invasion, replication, and egress within the infected host (10, 13). Consequently, identifying unique parasite factors that are required for the invasion, replication, or egress processes could open new opportunities to curb toxoplasmosis (10, 13).

Protein kinases are a family of proteins that regulate nearly all biological processes in eukaryotic cells and have been exploited as drug targets in many disease contexts (14). In *Toxoplasma*, kinases have been shown to play key roles in parasite motility, invasion, replication, egress processes, and promotion of parasite survival within the host by nullifying host defense mechanisms (15–21). The *Toxoplasma* genome contains over 150 kinases that belong to nine groups of eukaryotic protein kinases that include AGC kinases, CAMK kinases, CK1 kinases, CMGC kinases, STE kinases, ROPK kinases, FIKK kinases, tyrosine kinase-like kinases (TKLs) kinases, and the atypical kinase group (22, 23). The TKLs share sequence homology with tyrosine kinases but can phosphorylate not only tyrosine residues but also serine and threonine. The *Toxoplasma* genome encodes eight TKL proteins, of which six have been suggested to be important for tachyzoite growth *in vitro* based on a genome-wide fitness screen (24). Our group is interested in defining the role of TKL kinases in *Toxoplasma* biology and pathogenesis. We have named the TKLs in *Toxoplasma* according to the fitness score with the largest contributor to parasite fitness being TgTKL1 and the one with the lowest fitness score for contributor to parasite fitness being TgTKL8 (25). As a first step toward characterizing these kinases, our group initially focused on TgTKL1, identifying it as a plant-like nuclear kinase that regulates genes required for parasite invasion into host cells and a crucial virulence factor in the mouse model (25).

In this study, we focused on TgTKL4, another member of the TKL family in *Toxoplasma* that has also been suggested to support parasite growth *in vitro* (24). Our findings revealed that TgTKL4 is a cell cycle-regulated kinase with dynamic localization in the parasite. Specifically, expression of TgTKL4 appears to be restricted to the synthesis (S) and mitosis-cytokinesis (M/C) phases, suggesting that this kinase could be important for parasite division. Accordingly, parasites deficient in TgTKL4 showed defects in lytic cycle stages, including replication and invasion. Importantly, loss of TgTKL4 results in parasites with abnormal morphology associated with defects in the arrangement of subpellicular microtubules. Additionally, quantitative phospho-proteomics analysis revealed that loss of TgTKL4 kinase results in significant changes in the parasite phospho-proteome, with many of the cytoskeletal proteins being less phosphorylated in the TgTKL4 null mutant. Furthermore, TgTKL4-deficient parasites showed attenuated virulence in the mouse model, suggesting that this kinase is an important virulence factor. Together, these results established that TgTKL4 is a novel kinase that plays a key role in determining parasite morphology important for optimal parasite propagation *in vitro* and virulence *in vivo*.

## RESULTS

### TgTKL4 is a cell cycle-regulated kinase with dynamic localization in the parasite.

**FIG 1:**
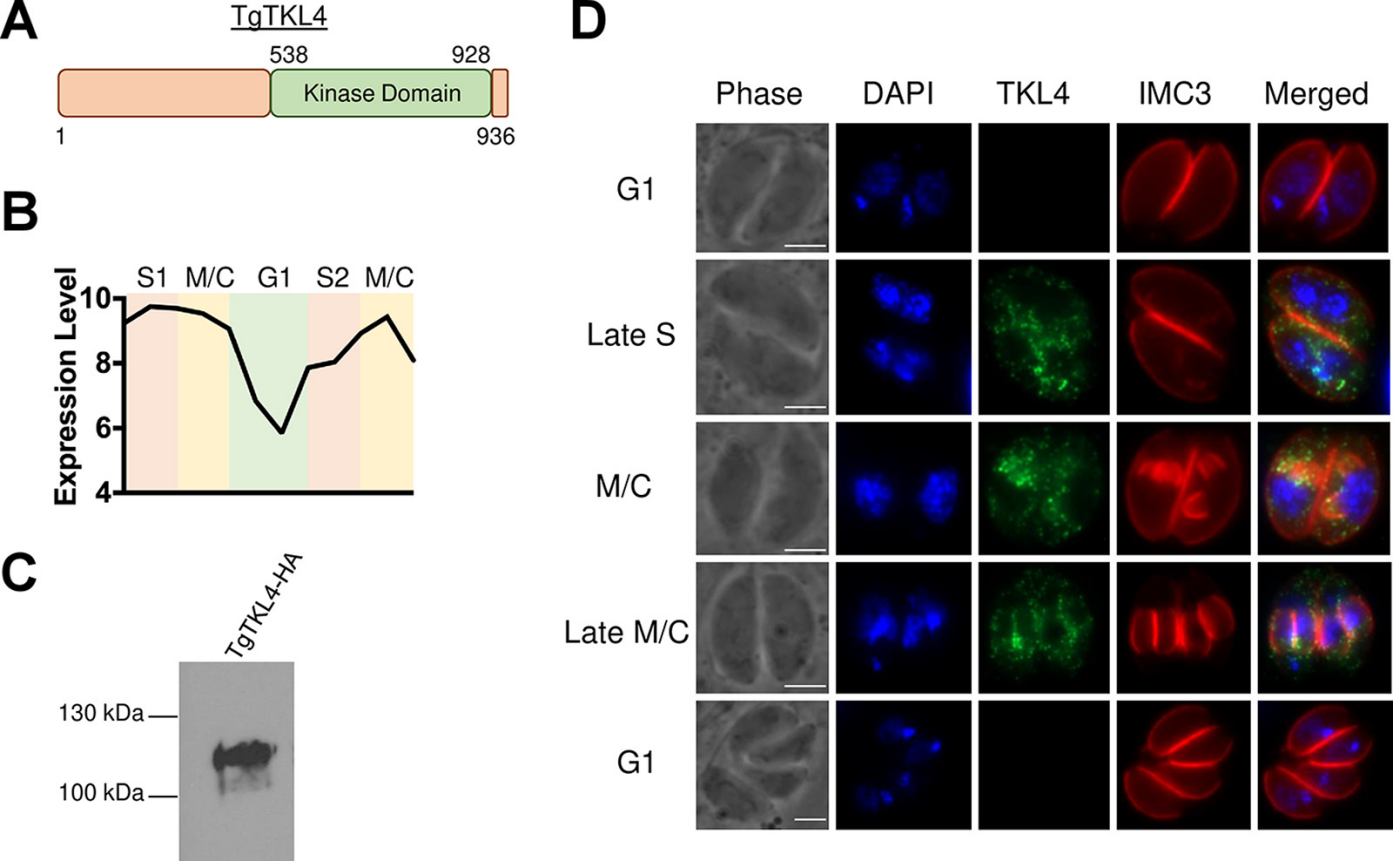
TgTKL4 is a cell cycle-dependent kinase. (A) Schematic representation of TgTKL4 domain architecture, with relative localization of kinase domain. (B) Gene expression profile of TgTKL4 in different stages of the cell cycle (26). (C) Western blot analysis of TgTKL4-HA parasites with anti-HA antibody. (D) Localization of TgTKL4 in intracellular parasites at different stages of the cell cycle using anti-HA antibody. IMC3 that localizes to the pellicle was used as a marker for different cell cycle stages. Scale bar, 2 μm.

The *Toxoplasma* genome contains eight TKL kinases, and six of these have been suggested to be important for parasite growth *in vitro*, including TgTKL4 (24). TgTKL4 is 936 amino acids in length, and the kinase domain is in the C-terminal region of the protein (Fig. 1A). A previous cell cycle transcriptome analysis suggested S and M/C phase expression of TKL4 (26) (Fig. 1B), implying that TgTKL4 expression is regulated transcriptionally. To determine the localization and also cell cycle regulation of TgTKL4 in *Toxoplasma*, we introduced a 3× hemagglutinin (HA) epitope tag at the 3′ end of the endogenous gene (27). Western blot analysis using an anti-HA antibody revealed a single band of the expected size in the endogenously HA-tagged clone (Fig. 1C). Immunofluorescence analysis using an anti-HA antibody showed that the expression of this kinase was indeed temporally regulated, with TgTKL4 being visualized only during the S and M/C phases, whereas the protein was not detected in the G_1_ phase of the cell cycle (Fig. 1D). In addition, TgTKL4 showed varied localization in the parasite during different stages of the cell cycle. In the late S phase, TgTKL4 appeared to be cytoplasmic, whereas in the late M/C phases, a fraction of the protein appeared to localize to the inner membrane complex (IMC) of the newly forming daughter cells, in addition to being cytosolic (Fig. 1D). Thus, these findings showed that TgTKL4 is a temporally and spatially regulated kinase in *Toxoplasma*.

### TgTKL4 kinase is required for efficient parasite growth *in vitro*.

We next sought to determine the role of TgTKL4 in the lytic cycle biology of *Toxoplasma.* Towards this goal, we generated TgTKL4 knockout (TgTKL4-KO) and TgTKL4 complemented (TgTKL4-COM) strains, as described in Materials and Methods. Both the TgTKL4-KO and TgTKL4-COM strains were validated for correct integration by PCR and sequencing (see Fig. S1A and B and Fig. S2 in the supplemental material).

10.1128/msphere.00649-22.1 FIG S1 Generation of the TgTKL4-knockout strain. (A) Schematic showing the strategy used to delete the TgTKL4 gene. The locations of the primers used for validation of the TgTKL4 null mutant are also indicated. (B) PCR products amplified from genomic DNA samples of the parental and knockout strains. The size of the PCR amplicon is indicated on the left side of the gel image. Download FIG S1, TIF file, 0.2 MB. Copyright © 2023 Montano et al. 2023 Montano et al. https://creativecommons.org/licenses/by/4.0/ This content is distributed under the terms of the Creative Commons Attribution 4.0 International license.

10.1128/msphere.00649-22.2 FIG S2 Generation of the TgTKL4-complemented strain. (A) Schematic showing the strategy used in generating the TgTKL4-complemented strain. The location of the primers used for validation of the complemented clone are also indicated. Scissor icons show approximate CRISPR-Cas9 cleavage points on HXGPRT. (B) PCR products amplified from genomic DNA samples of the knockout and the complemented strains. The size of the PCR amplicon is indicated on the left side of the gel image. Download FIG S2, TIF file, 0.1 MB. Copyright © 2023 Montano et al. 2023 Montano et al. https://creativecommons.org/licenses/by/4.0/ This content is distributed under the terms of the Creative Commons Attribution 4.0 International license.

**FIG 2:**
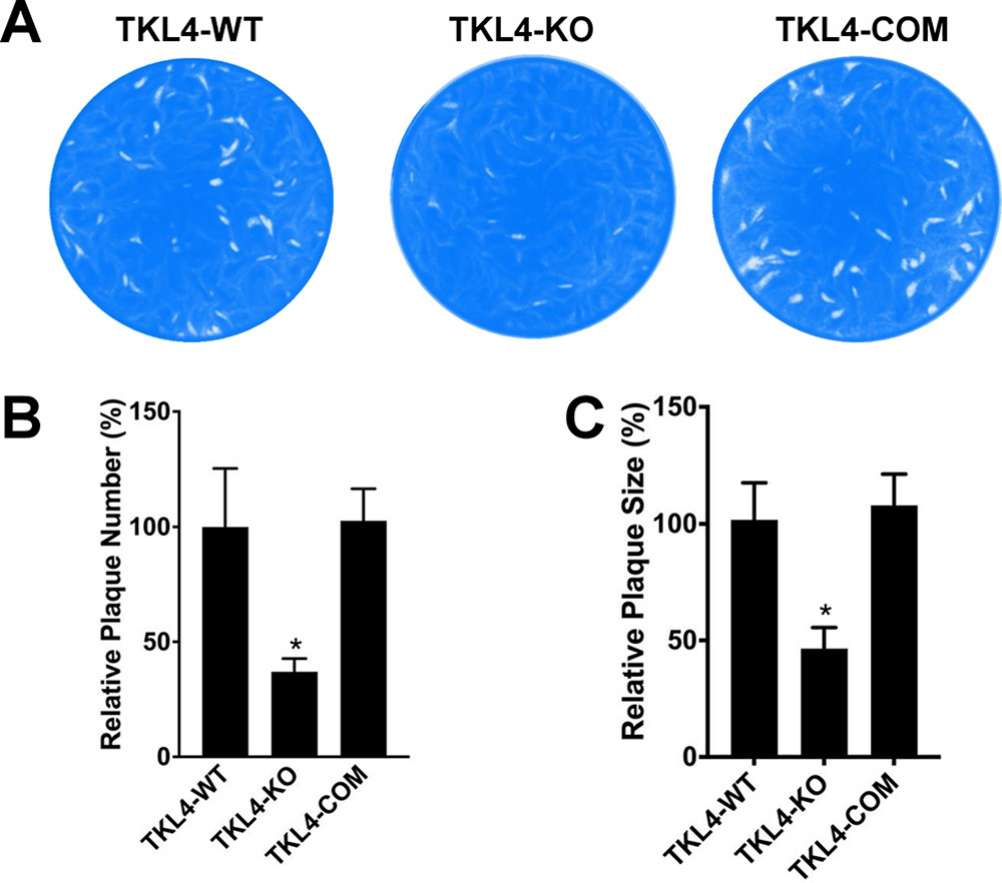
TgTKL4-knockout parasites showed defects in plaque formation. (A) Plaque assay examining the growth of wild-type (TgTKL4-WT), knockout (TgTKL4-KO), and complemented (TgTKL4-COM) parasites in HFF cells. Plaques are visible as clear zones on the background of a crystal violet-stained HFF monolayer. (B and C) Quantification of plaque number (B) and sizes (C) of TgTKL4-WT, TgTKL4-KO, and TgTKL4-COM parasites. Data were compiled from three independent experiments, and error bars represent standard errors of the means (SEM). *, *P < *0.05 (one-way analysis of variance [ANOVA]).

With these reagents in hand, we next examined the effect of the loss of TgTKL4 on parasite growth *in vitro* by monitoring the formation of plaques that resulted from multiple rounds of parasite invasion, replication, and egress on a confluent human foreskin fibroblasts (HFF) monolayer. We observed not only a significant (~63%) reduction in the number of plaques but also a significant (~45%) reduction in the sizes of the plaques formed by TgTKL4-KO parasites compared to TgTKL4-WT parasites (Fig. 2). Importantly, these reductions in plaque size and number were restored in the TgTKL4-complemented parasite strain (Fig. 2). These results indicated that TgTKL4 plays a critical role in the *Toxoplasma* tachyzoite lytic cycle *in vitro*.

### TgTKL4-deficient parasites showed defects in host cell invasion and parasite replication.

**FIG 3:**
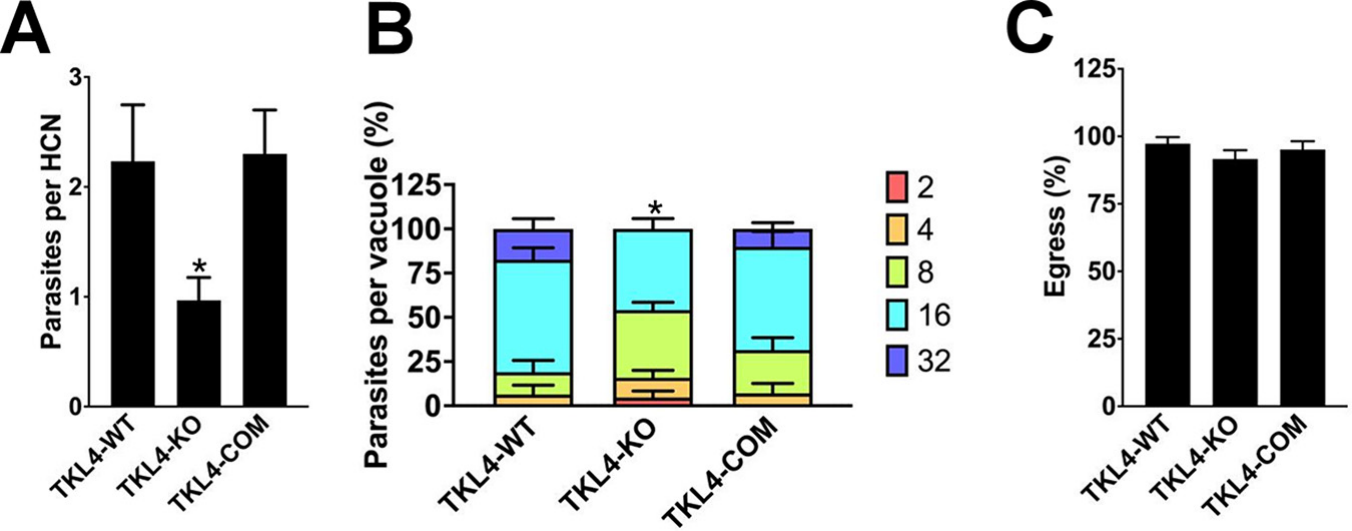
TgTKL4-deficient parasites showed defects in host cell invasion and replication. The wild-type (TgTKL4-WT), knockout (TgTKL4-KO), and complemented (TgTKL4-COM) parasites were subjected to lytic cycle assays, including invasion (A), replication (B), and induced egress (C) assays. Data were compiled from three independent experiments, and error bars represent SEM. *, *P < *0.05 (one-way ANOVA).

An impairment of plaque formation can be caused by defects in one or more steps of the parasite lytic cycle. Therefore, we next sought to determine which aspect of the lytic cycle was impaired in TgTKL4-KO parasites. We first performed a parasite invasion assay with host cells, and the results revealed that there was a significant reduction (~59%) in the number of invading parasites in TgTKL4-KO parasites compared to levels in the TgTKL4 wild type (WT) (Fig. 3A). Importantly, invasion competence was restored in the TgTKL4-COM strain (Fig. 3A), suggesting that TgTKL4 is important for the *Toxoplasma* invasion process.

We next assessed parasite replication using standard doubling assays (23), and we observed that at 30 h postinfection, TgTKL4-KO parasites contained fewer parasites per vacuole than the TgTKL4-WT strain. Further, the defect in replication was restored in the TgTKL4-COM strain, suggesting that TgTKL4 is required for efficient intracellular growth of *Toxoplasma* (Fig. 3B). Finally, we performed ionophore-induced egress assays using the Ca^2+^ ionophore A23187. However, we did not see statistically significant differences in the egress capability of TgTKL4-KO mutants compared to the wild-type or the complemented strain (Fig. 3C), suggesting that TgTKL4 does not play a role in *Toxoplasma* egress from the host cells. Overall, these findings indicated that TgTKL4 is important for *Toxoplasma* invasion into host cells and intracellular growth.

### TgTKL4-deficient parasites exhibited abnormal features in intracellular stages and deviant morphology in extracellular stages.

**FIG 4:**
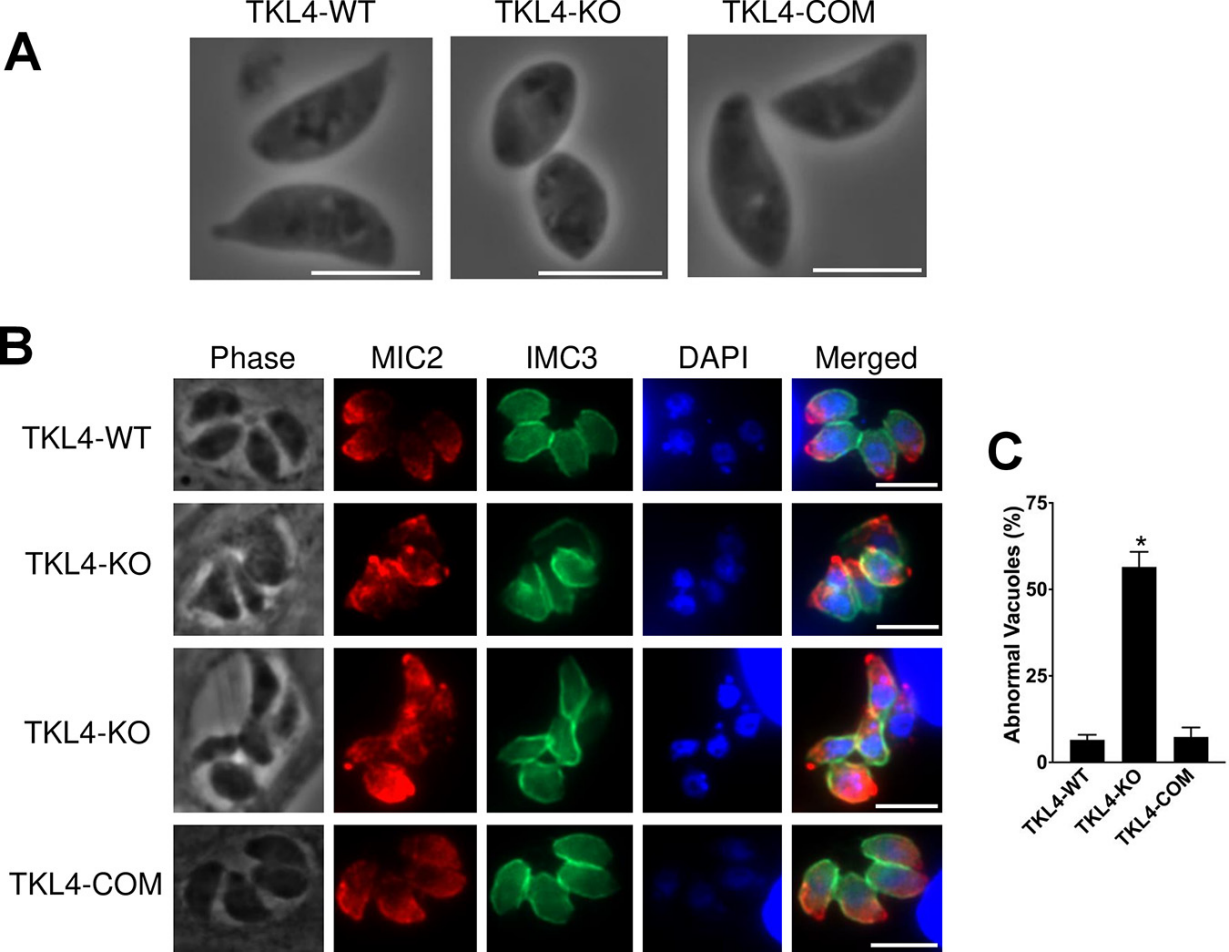
TgTKL4-deficient parasites exhibited abnormal features in intracellular stages and deviant morphology in extracellular stages. (A) TgTKL4-deficient parasites displayed a misshapen appearance in extracellular stages. (B) Loss of TgTKL4 resulted in vacuoles containing parasites with distorted shapes and odd numbers. Antibodies against IMC3 and MIC2 were used to stain dividing intracellular parasites. (C) Quantification of abnormal vacuoles in wild-type, knockout, and complemented strains. *n* = 3 independent experiments. Error bars represent SEM. *, *P < *0.05 (one-way ANOVA).

Interestingly, during our routine parasite culturing, we noticed that parasites deficient in TgTKL4 kinase show morphological features that were quite deviant from that of wild-type parasites. In extracellular stages, the TgTKL4 null mutants appeared misshapen in comparison to the parental strain. More specifically, TgTKL4 null mutant parasites appeared shorter and rounder than wild-type parasites (Fig. 4A). In intracellular stages, compared to wild-type parasites, which were elongated and present in even numbers (i.e., 2, 4, and 8, etc., per vacuole), TgTKL4-KO parasites exhibited distorted shapes and were often seen with an uneven number of parasites per vacuole (Fig. 4B and C). Normal morphology was restored in the complemented strain both in extracellular and intracellular forms (Fig. 4A and B). Together, these findings suggested that TgTKL4 plays an important role in *Toxoplasma* endodyogeny.

**FIG 5:**
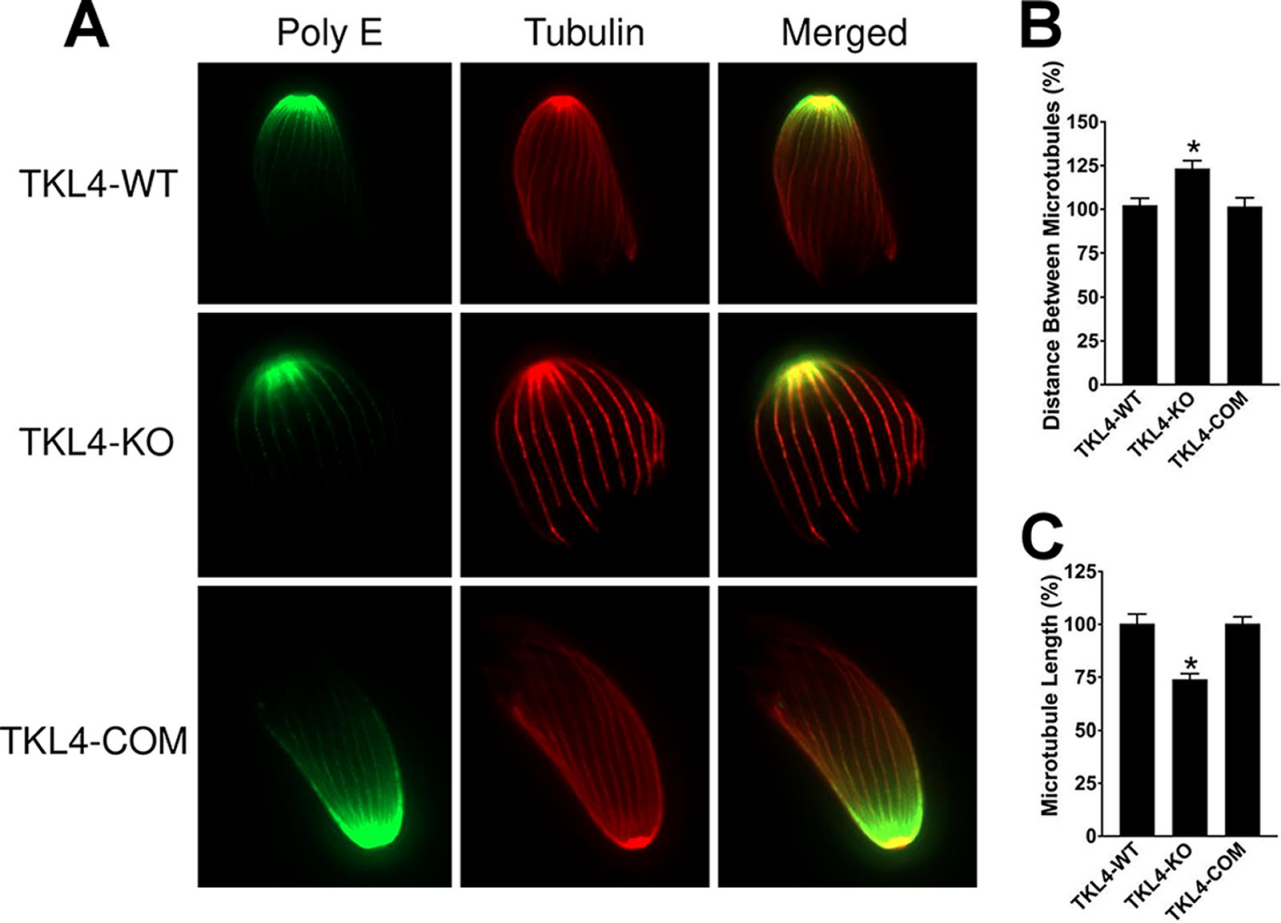
Loss of TgTKL4 affects subpellicular microtubule arrangement in *Toxoplasma*. (A) U-ExM analysis of wild-type (TgTKL4-WT), knockout (TgTKL4-KO), and complemented (TgTKL4-COM) strains using anti-polyglutamate chain (polyE) and anti-acetylated α-tubulin antibodies (41). (B and C) Quantification of the length and space between subpellicular microtubules in wild-type (TgTKL4-WT), knockout (TgTKL4-KO), and complemented (TgTKL4-COM) strains. Data were compiled from three independent experiments, and error bars represent SEM. *, *P < *0.05 (one-way ANOVA).

Previous studies have shown that subpellicular microtubules are one of the major determinants of parasite shape (28). Hence, to determine if the abnormal morphology in TgTKL4-deficient parasites was due to a defect in subpellicular microtubules, we performed ultrastructure expansion microscopy (U-ExM) (29). The results revealed that microtubules had altered appearance in TgTKL4 null mutants compared to the wild type or the complemented strain (Fig. 5). More specifically, the distance between the microtubules was increased in TgTKL4-deficient parasites (Fig. 5A and B). In addition, the length of the microtubules was shorter than in the parental or the complemented strain (Fig. 5A and C). Furthermore, whereas microtubules of wild-type and complemented parasites had a spiral appearance, the TgTKL4 null mutants displayed straight or nonspiral microtubules (Fig. 5A).

### TgTKL4-deficient parasites showed altered transcriptomes.

**FIG 6:**
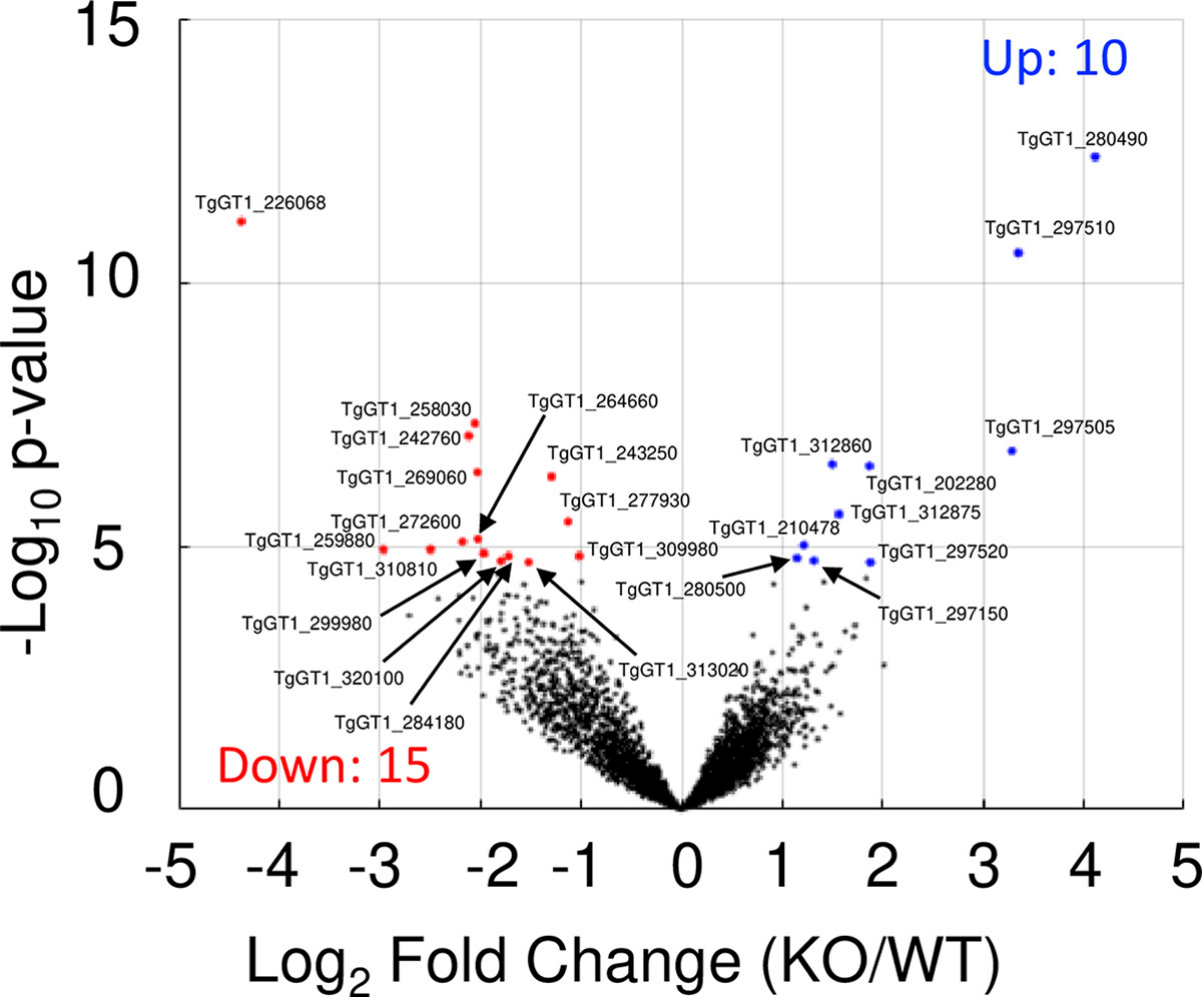
Volcano plot of statistical significance (−log_10_ *P* value) versus fold change (log_2_), highlighting genes identified as differentially expressed (FDR, ≤0.005) in TgTKL4-KO parasites compared to TgTKL4-WT parasites. Downregulated genes (15 genes) are highlighted in red, and upregulated genes (10 genes) are highlighted in blue.

Next, we wanted to determine if loss of TgTKL4 would result in changes in the gene expression profile in the null mutant strain. To test this, we purified RNA from TgTKL4-WT and TgTKL4-KO parasites and performed transcriptomics analysis to assess the effects of loss of TgTKL4 on gene expression globally. The RNA sequencing studies showed that there were only 25 genes that were significantly differentially expressed (log_2_ fold change, ≥1, false-discovery rate [FDR], ≤0.005) in the TgTKL4-KO mutant compared to TgTKL4-WT parasites (Fig. 6; see also Data Set S1). Of these 25 differentially expressed genes, 15 genes were downregulated in TgTKL4-KO parasites, while 10 genes were upregulated. We next manually assigned functional classifications for the 25 differentially expressed genes based on their known or putative functions (Data Set S1). This revealed that the differentially regulated gene set contained genes involved in protein trafficking, metabolism, host cell invasion, signaling, gene expression, and DNA replication. Together, these findings suggested that loss of TgTKL4 resulted in only moderate changes in gene expression.

10.1128/msphere.00649-22.4 DATA SET S1 List of genes dysregulated in TgTKL4-knockout parasites. Download Data Set S1, XLSX file, 0.01 MB. Copyright © 2023 Montano et al. 2023 Montano et al. https://creativecommons.org/licenses/by/4.0/ This content is distributed under the terms of the Creative Commons Attribution 4.0 International license.

### Loss of TgTKL4 caused global changes in the parasite phospho-proteome.

**FIG 7:**
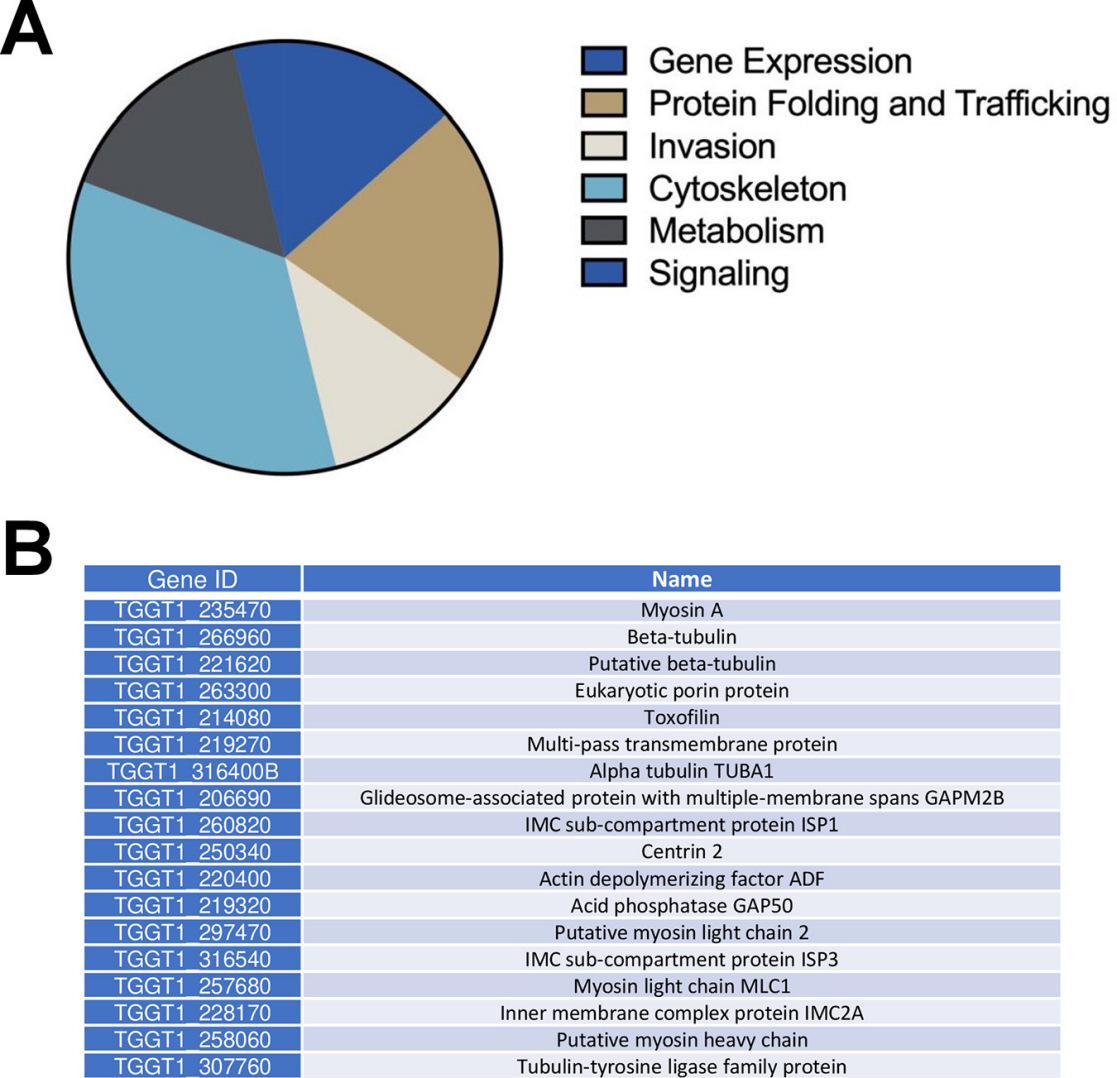
Loss of TgTKL4 results in dysregulation of the phospho-proteome in *Toxoplasma*. (A) Pie chart showing different categories of genes that are less phosphorylated in TgTKL4 null mutants than in wild-type (TgTKL4-WT) and complemented (TgTKL4-COM) strains. (B) List of cytoskeletal proteins that were less phosphorylated in TgTKL4 null mutants than in wild-type (TgTKL4-WT) or complemented (TgTKL4-COM) strains.

The TgTKL4 kinase domain contains all the conserved motifs required for catalytic activity, rendering it likely that TgTKL4 is a functional kinase. Having found minimal changes in gene expression at the transcript level, we reasoned that phenotypes exhibited by TgTKL4-KO parasites could be due to changes in protein phosphorylation. To determine phosphorylation changes that occurred in the absence of TgTKL4, we performed quantitative phospho-proteome analysis of wild-type, TgTKL4-KO, and TgTKL4-COM strains. This analysis identified 52 proteins that were less phosphorylated in TgTKL4-KO parasites than in the parental or the complemented strains. The proteins that showed decreased phosphorylation in TgTKL4-KO parasites have been shown or predicted to be involved in different functions, including gene expression, protein folding and transport, invasion, metabolism, signaling, and cytoskeletal constituents (Fig. 7A; Data Sets S2 and S3). Interestingly, proteins involved in building the parasite cytoskeleton constituted a large fraction of the set that showed reduced phosphorylation in TgTKL4 null mutants (Fig. 7A and B). It is feasible that these proteins could be direct substrates of TgTKL4 or these results could be due to downstream effects of the absence of TgTKL4. Together, these findings suggested that loss of TgTKL4 results in significant changes in the *Toxoplasma* phospho-proteome, with many of these changes occurring in cytoskeletal proteins.

10.1128/msphere.00649-22.5 DATA SET S2 List of proteins less phosphorylated in TgTKL4-knockout parasites. Download Data Set S2, XLSX file, 0.01 MB. Copyright © 2023 Montano et al. 2023 Montano et al. https://creativecommons.org/licenses/by/4.0/ This content is distributed under the terms of the Creative Commons Attribution 4.0 International license.

10.1128/msphere.00649-22.6 DATA SET S3 MS data of the TgTKL4 phospho-proteome. Download Data Set S3, XLSX file, 3.3 MB. Copyright © 2023 Montano et al. 2023 Montano et al. https://creativecommons.org/licenses/by/4.0/ This content is distributed under the terms of the Creative Commons Attribution 4.0 International license.

### TgTKL4 is important for parasite virulence in mice.

**FIG 8:**
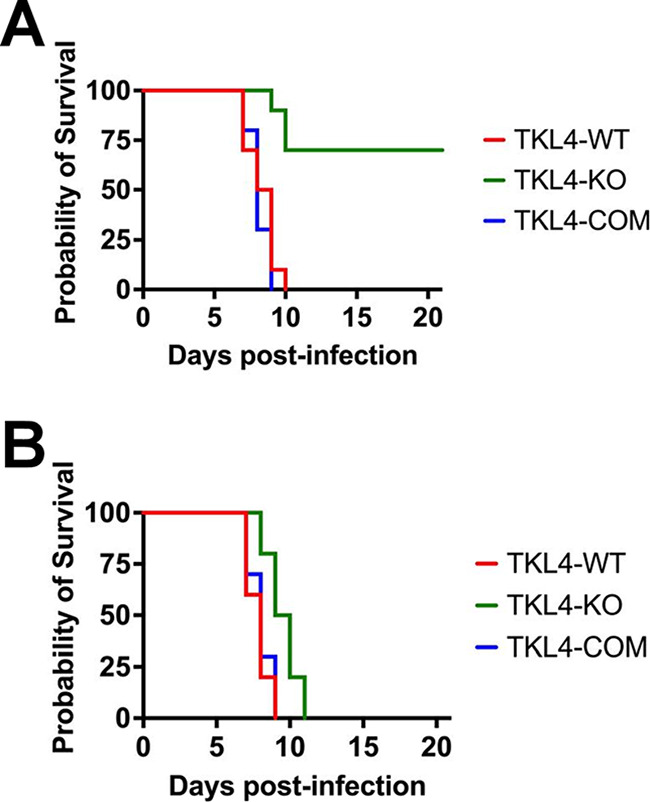
Loss of TgTKL4 attenuates parasite virulence *in vivo*. Mice were injected intraperitoneally with 20 tachyzoites (A) or 100 tachyzoites (B) of either wild-type (TgTKL4-WT), knockout (TgTKL4-KO), or complemented (TgTKL4-COM) strains. (The data were pooled from two independent experiments, and in each experiment 5 mice per strain were inoculated). Concomitant plaque assays were performed to corroborate the numbers of viable tachyzoites injected. The data were analyzed using a log-rank (Mantel-Cox) test. *P <* 0.0001 (A); *P < *0.0016 (B).

The global screen aimed at identifying genes important for parasite fitness suggested that TgTKL4 is important for parasite fitness. However, it is not known if TgTKL4 is important for parasite pathogenesis *in vivo*. Hence, to determine the role of TgTKL4 in *Toxoplasma* pathogenesis in an animal model, 10 female CBA/J mice were infected with either 20 or 100 parasites of TgTKL4 wild-type, knockout, or complemented parasites via intraperitoneal injection. Remarkably, while all of the mice injected with 20 tachyzoites of either wild-type or complemented parasites died within 10 days of infection, 70% of the mice injected with TgTKL4 knockout parasites survived (Fig. 8A). Analysis of serum samples from the survivor mice at 3 weeks postinfection confirmed parasite exposure, as all mice were found to be seropositive (data not shown). However, when the parasite number was increased to 100 tachyzoites per inoculation, although all mice injected with all three strains died, TgTKL4-KO-inoculated mice succumbed to infection with delayed kinetics compared to responses in mice infected with wild-type or complemented strains (Fig. 8B). Together, these results suggested that loss of TgTKL4 leads to attenuation of parasite virulence, and hence TgTKL4 is an important factor for *Toxoplasma* pathogenesis in the animal model.

## DISCUSSION

Protein kinases are an important class of signaling enzymes that have been shown to regulate numerous aspects of eukaryotic cell biology, including protozoan parasites. In this study, we sought to characterize TgTKL4, a member of the newly described TKL family kinases in *Toxoplasma*. Since the CRISPR-based genome-wide screen aimed at identifying genes important for parasite fitness suggested that TgTKL4 is important for *Toxoplasma* propagation, we further wanted to understand the role of this kinase in parasite biology. Our findings showed that TgTKL4 is a cell cycle-regulated kinase that predominantly localizes to the cytoplasm in the S phase and to the IMC region during daughter cell formation. The loss of TgTKL4 results in defects in parasite morphology along with reduced competence in replication and invasion events. Using expansion microscopy, we showed that one of the major causes of abnormal morphology in TgTKL4-deficient parasites was due to changes in the arrangement of subpellicular microtubules. To identify putative substrates of TgTKL4, we performed quantitative phospho-proteome analysis of the wild type and TgTKL4 null mutants, and the findings revealed that many of the cytoskeletal proteins were indeed less phosphorylated in the absence of TgTKL4. In addition, loss of TgTKL4 also reduced parasite virulence in the animal model, suggesting that the kinase is important for parasite pathogenesis.

It is interesting that the expression of TgTKL4 is confined to certain stages in the cell cycle, although the effects of its absence are observed in other stages as well. This could be due to either a direct effect on TgTKL4 function or downstream effects of TgTKL4 signaling pathway. Although it appears that expression of TgTKL4 is controlled at the transcriptional level, the *cis*-acting elements involved in stage-specific expression of this kinase remain undetermined. Future studies focused on dissecting the TgTKL4 promoter could throw more light on the DNA motifs and transcription factors involved in this process.

Our findings that showed TgTKL4 is required for efficient growth of *Toxoplasma* were in alignment with the CRISPR-based phenotypic score (24). Since TgTKL4 is expressed in the S and M/C phases, it was not very surprising that loss of TgTKL4 resulted in moderate defects in parasite replication. Although TgTKL4-deficient parasites had a defect in host cell invasion, it is unlikely that this kinase has a direct role in this process. Since TgTKL4 is expressed only during endodyogeny and is not expressed in the G_1_ stage of the cell cycle, the defect in invading host cells was mostly due to indirect effects of abnormal cytoskeleton and morphology. Similarly, the attenuated virulence of TgTKL4-deficient parasites in the animal model was also most likely due to defects in parasite fitness.

The cortical cytoskeleton of *Toxoplasma*, also known as the pellicle, is a complex layered structure that includes parasite plasma membrane on the outside and the IMC beneath it (10, 30). Further, the IMC region itself is comprised of double-membraned alveoli on the top and a subpellicular network (made of intermediate filament-like proteins) in the bottom and is supported by the subpellicular microtubules. There are 22 subpellicular microtubules in *Toxoplasma* that are very stable and extend from the apical polar ring to about two-thirds of the length of the parasite (8, 30). Previous studies have shown that subpellicular microtubules are one of the major determinants of parasite shape (28, 31, 32).

In parasites deficient in TgTKL4, the arrangement of these subpellicular microtubules was perturbed and, consequently, the TgTKL4-KO parasites appeared misshapen. However, the dynamic localization of TgTKL4 during intracellular stages was more interesting. Our studies showed that TgTKL4 relocalizes from the cytoplasm to the IMC region during daughter cell budding and that absence of this kinase affects cytoskeletal arrangement. Previous studies have also shown that IMC proteins and, more specifically, the glideosome-associated protein with multiple-membrane spans (GAPM) family of proteins, play a critical role in stability of the cortical microtubules (28). Interestingly, our phospho-proteome analysis of TgTKL4 mutants indicated that many of the cytoskeletal proteins, including IMC proteins, were less phosphorylated, suggesting that these could be putative substrates. Hence, it is feasible that posttranslational modification of proteins involved in building daughter cells by TgTKL4 is a key event that dictates parasite morphology. However, more studies are needed to precisely identify the true substrates of TgTKL4 and how these phosphorylation events result in cytoskeletal modification and consequently parasite morphology.

## MATERIALS AND METHODS

### Parasite cultures.

Toxoplasma gondii tachyzoites were maintained by passage through HFF in a humidified incubator at 37°C with 5% CO_2_. Normal growth medium consisted of Dulbecco’s modified Eagle’s medium (DMEM) supplemented with 10% fetal bovine serum, 2 mM l-glutamine, and 50 μg/mL of penicillin-streptomycin. Purification of parasites was performed as previously described (33).

### Immunofluorescence microscopy.

Immunofluorescence staining of intracellular parasites was performed according to a previously described procedure (34). The primary antibodies used were mouse anti-HA (Cell Signaling Technology catalog number 6E2; 1:1,000), rabbit anti-HA (Cell Signaling Technology catalog number C29F4; 1:1,000), and rabbit anti-IMC6 (1:1,000) and rabbit anti-IMC3 (1:2,000). Secondary antibodies used included Alexa Fluor 594- or Alexa Fluor 488-conjugated goat anti-rabbit or goat anti-mouse antibodies (Molecular Probes; 1:1,000). Slides were viewed using a Zeiss Axio Observer 7 microscope (Carl Zeiss), and digital images were captured with an Axiocam 506 mono charge-coupled-device camera using Axiovision software.

### Generation of TgTKL4 knockout and complemented strains.

A TgTKL4 knockout plasmid construct was made by inserting approximately 1-kb regions of sequence homology corresponding to regions 5′ and 3′ of the TgTKL4 coding sequence into the pminiHXGPRT plasmid (35), using the primers listed in Table S1. Briefly, the TgTKL4 5′ flank was amplified and directionally cloned into KpnI- and HindIII-digested pminiHXGPRT to generate TgTKL4.5′-HXGPRT. Similarly, the 3′ flank of TgTKL4 was amplified and directionally cloned into BamHI- and NotI-digested TgTKL4.5′-HXGPRT to generate the final TgTKL4 knockout construct. Prior to transfection, the DNA fragment containing the hypoxanthine-xanthine-guanine-phosphoribosyl transferase (HXGPRT) expression cassette flanked by the TgTKL4 5′ and 3′ homology regions was excised by digestion with KpnI and NotI, gel purified, and electroporated into the RHΔ*ku80* strain. The parasites were then cultured in the presence of 50 μg/mL mycophenolic acid and 50 μg/mL xanthine to select drug-resistant parasites and cloned by limiting dilution (36). The clones were screened and validated by PCR and sequencing.

10.1128/msphere.00649-22.3 TABLE S1 List of primers used in this study. Download Table S1, PDF file, 0.02 MB. Copyright © 2023 Montano et al. 2023 Montano et al. https://creativecommons.org/licenses/by/4.0/ This content is distributed under the terms of the Creative Commons Attribution 4.0 International license.

To obtain a TgTKL4 complemented parasite cell line, we amplified the TKL4 gene, including 1 kb of 5′ and 3′ flanking regions, using *Toxoplasma* genomic DNA as the template. We also generated CRIPSR-Cas9 plasmid with single guide RNA (sgRNA) sequences that would cut the HXGPRT gene using a Q5 site-directed mutagenesis kit (37, 38). The two sgRNAs that cut the HXGPRT after the start codon and near the stop codons were transfected along with the TKL4 gene, and parasites were cultured in the presence of 6-thioxanthine to select for the loss of HXGPRT. The parasites were then examined by sequencing and quantitative real-time PCR to confirm the expression of TgTKL4. The primers used in generating the complemented strain and sgRNA plasmids are listed in Table S1.

### Plaque assays.

Intracellular parasites were harvested, syringe filtered, and added onto a confluent monolayer of HFF cells in a 12-well plate (500 tachyzoites per well). The plates were then incubated at 37°C for 6 days without any movement. The plates were then washed with phosphate-buffered saline (PBS), methanol fixed, and stained with 2% crystal violet to visualize regions of host cell disruption.

### Invasion assay.

Invasion assays were performed in eight-well chamber slides as described previously with the following modifications (39). Purified tachyzoites were added onto HFF monolayers (2 × 10^6^ parasites/well) and incubated at 37°C for 30 min. Slides were then washed three times to remove noninvaded parasites, fixed, blocked, and stained with mouse anti-SAG1 without permeabilization. After 1 h, slides were washed, permeabilized with 0.01% TX-100, and stained with rabbit anti-M2AP antibody. The slides were further washed and stained with the secondary antibodies Alexa Fluor 594-conjugated goat anti-mouse (Molecular Probes) and Alexa Fluor 488-conjugated goat anti-rabbit (Molecular Probes). After 1 h, slides were washed and mounted using Vectashield (with 4′,6-diamidino-2-phenylindole). Parasites that were both red and green were identified as extracellular (attached), whereas those that were green but not red were identified as intracellular (invaded). Images of 15 random fields of view within each well were captured at 600× magnification, and the total numbers of intracellular parasites and host cell nuclei were determined.

### Replication assay.

To assess the parasite doubling time, freshly egressed parasites were inoculated into confluent HFF monolayers in 12-well plates and allowed to invade for 2 h. The monolayers were then washed three times with medium to remove uninvaded parasites and incubated at 37°C. At 30 h postinfection, the cells were fixed with methanol and stained using Diff-Quik (Dade-Behring) according to the manufacturer's instructions. For each treatment, at least 100 vacuoles from three biological replicates were assessed for the number of parasites per vacuole.

### Ionophore-induced egress assay.

The efficiency of egress after calcium ionophore treatment was determined using established protocols (40). Briefly, freshly harvested parasites were added to a 24-well plate containing confluent HFFs at a multiplicity of infection of 1 and were incubated at 37°C for 30 h. To induce egress, intracellular parasites were washed with warm PBS, incubated at 37°C for 2 min in Hanks' balanced salt solution containing 1 μM calcium ionophore A23187, and fixed with 100% methanol. To visualize intact and lysed vacuoles, the cultures were stained using Diff-Quik (Dade-Behring) according to the manufacturer's instructions. Percent egress was determined by dividing the number of lysed vacuoles by the total number of vacuoles for a sample.

### Ultrastructure expansion microscopy.

The U-ExM was performed according to previously published protocols with a few modifications (29). Briefly parental, TgTKL4 knockout and complemented strains were harvested, pelleted, and resuspended in PBS. Parasites were then be allowed to settle on poly(lysine)-coated coverslips for 10 min at room temperature. The samples were then fixed with paraformaldehyde and incubated in a solution of formaldehyde and acrylamide (AA) in PBS for 5 h at 37°C prior to gelation in APS/Temed/monomer solution (19% sodium acrylate; 10% AA, 0.1% bis-AA in 10× PBS) for 1 h at 37°C. The denaturation was performed for 90 min at 95°C. Gels were then expanded overnight in water, and after shrinking in PBS, gels were stained for 3 h at 37°C with primary antibodies against acetylated α-tubulin (catalog number 6-11B-1, Santa Cruz Biotechnology), polyglutamate chain (polyE), pAb(IN105), catalog number AG-25B-0030, Adipogen; 1:500] followed by secondary antibodies (41). A second round of expansion was performed overnight in water before imaging the slides. Imaging was performed on a Zeiss inverted microscope using a 63×,1.4-numerical aperture oil objective and Axiovision software. In each experiment, at least 20 parasites were imaged for each strain, and data from three independent experiments were used for quantification and statistical analysis.

### RNA sequencing and differential gene expression analysis.

RNA sequencing was performed according to previously published protocols with some modifications (25). Total RNA from freshly egressed TgTKL4-WT and TgTKL4-KO parasites was isolated using the RNeasy kit (Qiagen). The quality of total RNA samples was verified by using a BioAnalyzer (Agilent), followed by digestion with DNase I (NEB). rRNA was removed using the Ribo-Zero rRNA removal kit (human/mouse/rat; Illumina). Sequencing libraries were then generated using the TruSeq RNA sample prep kit (v2; Illumina) according to the manufacturer’s protocol. Libraries were amplified using the TruSeq cluster kit (v3; Illumina) and subjected to 50-bp single-end sequencing with the Illumina HiSeq 2000 system. Sequencing reads were aligned to the *Toxoplasma* GT1 reference genome (ToxoDB v.53; www.toxodb.org) using the STAR software package (v.2.7.1a, with default settings) (42). Filtered and normalized gene expression levels were calculated from the aligned reads using HTSeq v.0.13.5 (43). Differentially expressed genes were identified by linear modeling and Bayesian statistics using the limma package for R v.3.49.1 (44).

### Quantitative phospho-proteomics.

Intracellular parasites were harvested, syringe filtered, and pelleted by centrifuging at 1,000 × *g* for 10 min. The parasite pellets were treated with Pierce universal nuclease (ThermoFisher Scientific) to reduce viscosity and prevent any competitive binding by oligonucleotides to the phosphopeptide enrichment materials. Cysteine disulfide bonds were reduced using dithiothreitol (DTT), free sulfhydryl groups were alkylated with iodoacetamide (IAA), and unreacted IAA was quenched with an excess of DTT. Samples were acidified using *o*-phosphoric acid, and protein was precipitated using liquid chromatography-mass spectrometry (LC-MS)-grade methanol and incubation at −80°C.

Precipitated protein was loaded onto S-Trap mini spin columns (Protifi) and digested using Pierce trypsin protease, MS grade (ThermoFisher Scientific). The resulting peptides were collected by centrifugation and dried. Peptides were solubilized and phosphopeptides were enriched by first using a High-Select TiO_2_ phosphopeptide enrichment kit followed by a High-Select Fe-nitrilotriacetic acid (NTA) phosphopeptide enrichment kit according to the directions in the manuals supplied with each kit. Phosphopeptides eluted from the Fe-NTA column were eluted into the same tube as those eluted from the TiO_2_ column, giving a single phosphopeptide sample for each original sample.

The phosphopeptide samples and flowthrough samples (material which did not interact with the phosphopeptide enrichment materials) were analyzed in duplicate using LC-tandem MS (MS/MS). LC-MS/MS was performed utilizing a Lumos Fusion Orbitrap (ThermoFisher Scientific), and data were analyzed using Proteome Discoverer 2.5 (ThermoFisher Scientific).

### *In vivo* virulence assays.

Six-week-old female CBA/J mice were injected intraperitoneally with either TgTKL4-WT, TgTKL4-KO, or TgTKL4-COM parasites (20 or 100 parasites in 100 μL PBS, five mice per strain). To verify the viability of the injected parasites, an equivalent number of parasites from the same preparation used for mouse injections was used to inoculate an HFF monolayer for plaque assays immediately following injection. The viability of both groups of the injected parasites was again confirmed using plaque assays as described above. Seroconversion of all surviving mice was confirmed with Western blot analysis using serum collected 3 weeks postinfection and RHΔ*ku80* parasite lysate.

### Ethics statement.

All laboratory animal work in this study was carried out in accordance with policies and guidelines specified by the Office of Laboratory Animal Welfare, U.S. Department of Agriculture, and the American Association for Accreditation of Laboratory Animal Care. The University of Michigan Committee on the Use and Care of Animals approved the animal protocol used for this study (Animal Welfare Assurance A3114-01, protocol PRO00008638).
