# Supplementary figures and images for: TgTKL4 Is a Novel Kinase That Plays an Important Role in Toxoplasma Morphology and Fitness
Source: mSphere. 2023 Feb 14;8(2):e00649-22. doi: 10.1128/msphere.00649-22 (PMC10117109; doi:10.1128/msphere.00649-22)

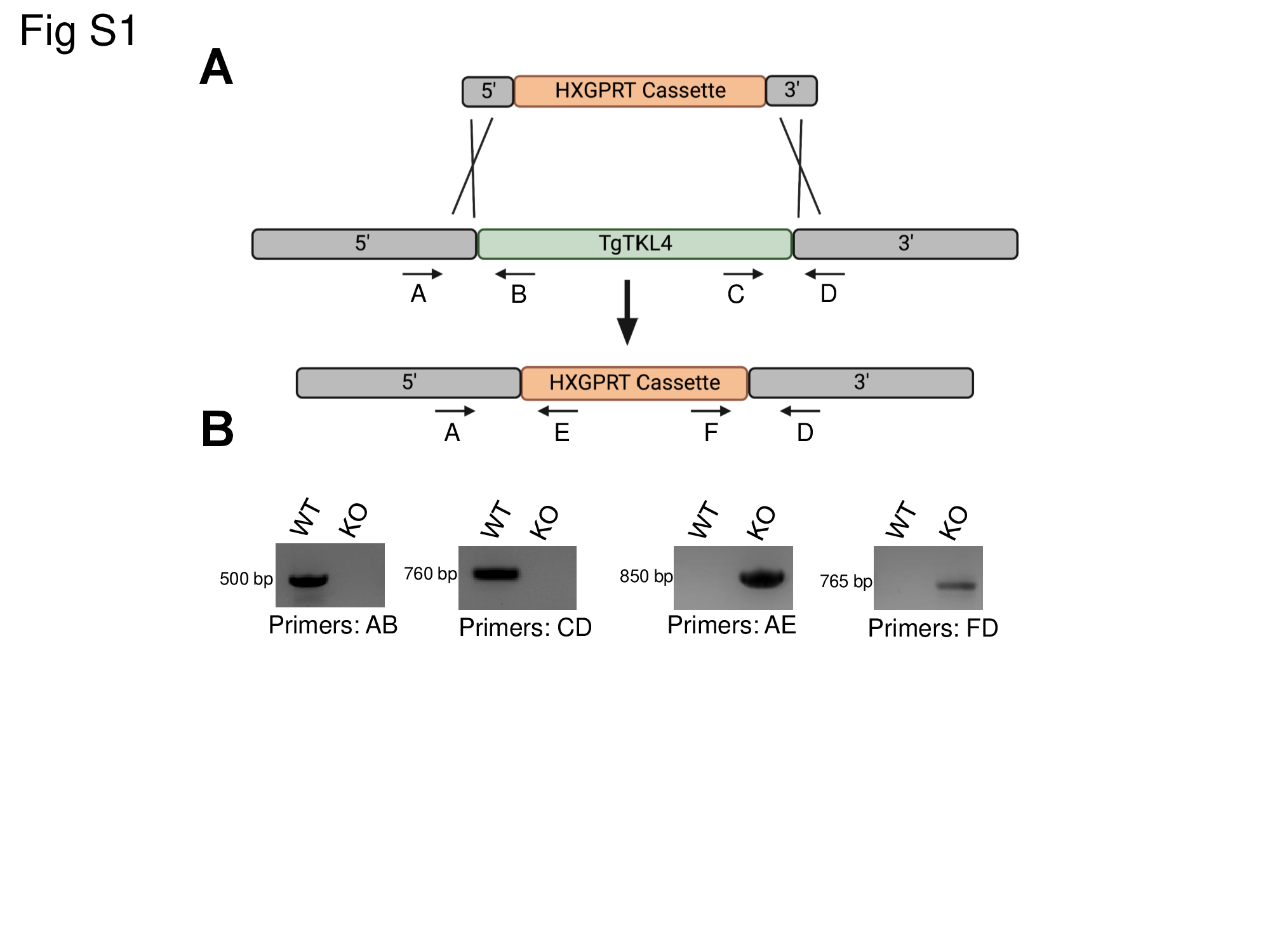

Supplement: FIG S1 [file msphere.00649-22-s0001.tif]

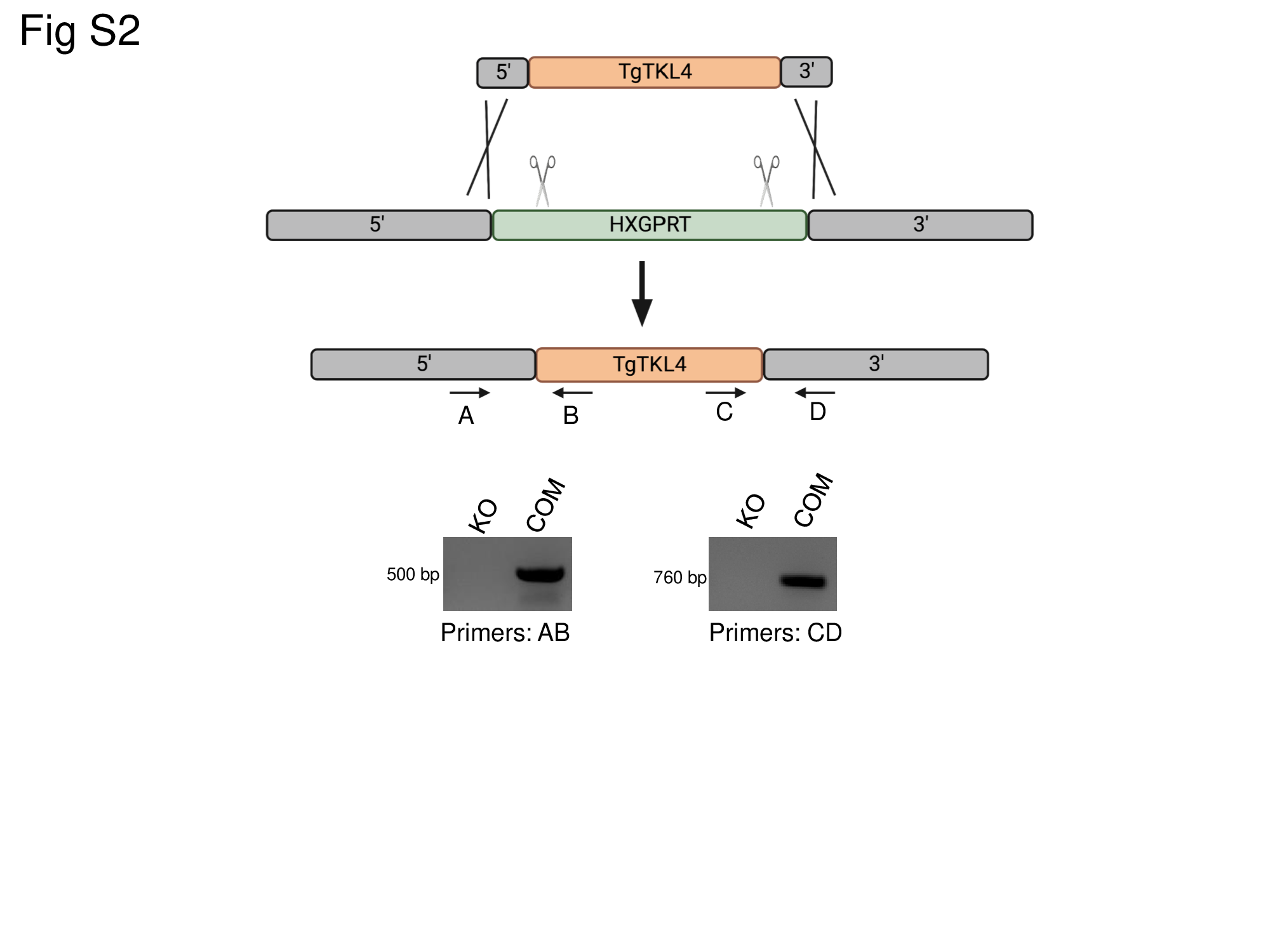

Supplement: FIG S2 [file msphere.00649-22-s0002.tif]
