# Supplementary material for: TgTKL4 Is a Novel Kinase That Plays an Important Role in Toxoplasma Morphology and Fitness
Source: mSphere. 2023 Feb 14;8(2):e00649-22. doi: 10.1128/msphere.00649-22 (PMC10117109; doi:10.1128/msphere.00649-22)
Supplement: TABLE S1 [file msphere.00649-22-s0003.pdf]

Table S1. Primers used in this study.

S. No	Name	Sequence
1	TgTKL4.IF.F	ttccaatccaatttaattaaGCTTCTTTGATTCACTTGATGACACGG
2	TgTKL4.IF.R	ccacttccaattttaattaaCAGTCCGTCGATGATGATCTCC
3	TgTKL4.KpnI.F	gatcGGTACCCCAGGAACATCTGTGACGCAAG
4	TgTKL4.HindIII.R	gatcAAGCTTTATGCAATGCAGCCGCGAAACG
5	TgTKL4.BamHI.F	gatcGGATCCGACGGCAGAGGAATTTGACAAG
6	TgTKL4.NotI.R	gatcGCGGCCGCCCATTAGTTCGTGTAGGTGGTC
7	TKL4.genDNA.KpnI.F	ACTGGGATCCTGACACTCGACCCATACTGAG
8	TKL4.genDNA.NotI.R	actgGCGGCCGCGAATCATGTCCTGACGGTAC
9	HX.SG1.F	caccgagttcGTTTTAGAGCTAGAAATAGC
10	HX.SG1.R	agggtgaaacAACTTGACATCCCCATTTAC
11	HX.SG2.F	tcgtcgacacGTTTTAGAGCTAGAAATAGC
12	HX.SG2.R	tgtcctcaacAACTTGACATCCCCATTTAC
13	TKL4.5.F1	TGACACTCGACCCATACTGAG
14	HXG.R1	catcagcgttgtagaaggtg
15	DHFR.3.F2	gttgacttgttaggctccgac
16	TKL4.3.R1	GAATCATGTCCTGACGGTAC
17	TKL4.5.R1	ACTCAGATCTACGGGTTCCGAG
18	TKL4.3.F1	TAAGCCTAGGGTATGTCCGGAC
